# Mechanism of interventional effect and targets of Zhuyu pill in regulating and suppressing colitis and cholestasis

**DOI:** 10.3389/fphar.2022.1038188

**Published:** 2022-11-02

**Authors:** Han Yu, Fenghua Zhang, Yueqiang Wen, Zhili Zheng, Gaoyang Chen, Yingying Pan, Peijie Wu, Qiaobo Ye, Jun Han, Xiaofeng Chen, Chao Liu, Tao Shen

**Affiliations:** ^1^ School of Basic Medicine, Chengdu University of Traditional Chinese Medicine, Chengdu, China; ^2^ Department of Pediatrics, Guang’an Hospital of Traditional Chinese Medicine, Guang’an, China; ^3^ Department of Traditional Chinese Medicine, Beijing University of Chinese Medicine, Beijing, China

**Keywords:** cholestasis, colitis, common interventional mechanism, fecal microbial diversity, fecal metabolism, Zhuyu pill

## Abstract

Zhuyu pill (ZYP) is a traditional Chinese medicine prescription composed of two drugs, *Coptis chinensis* Franch. and *Tetradium ruticarpum* (A. Jussieu) T. G. Hartley, and is commonly used in the clinical treatment of diseases of the digestive system. However, the mechanism underlying the effect of ZYP on colitis remains unclear. In this study, a colitis rat model was induced with 2,4,6-trinitro-benzenesulfonic acid (TNBS, 100 mg/kg) and treated with ZYP (low dose: 0.6 g/kg, high dose: 1.2 g/kg). Disease activity index, colonic weight index, and weight change ratio were used to evaluate the model and efficacy. LC-MS and 16S rRNA gene sequencing were used to measure differences in fecal metabolism and microorganism population among the control, model, low-dose ZYP, and high-dose ZYP groups. To elucidate the mechanism of interventional effect of ZYP, Spearman correlation analysis was used to analyze the correlation between fecal metabolism and fecal microbial number. High-dose and low-dose ZYP both exhibited significant interventional effects on colitis rat models, and high-dose ZYP produced a better interventional effect compared with low-dose ZYP. Based on a metabolomics test of fecal samples, significantly altered metabolites in the model and high-dose ZYP treatment groups were identified. In total, 492 metabolites were differentially expressed. Additionally, sequencing of the 16S rRNA gene in fecal samples revealed that the high-dose ZYP could improve TNBS-induced fecal microbiota dysbiosis. Ultimately, changes in tryptophan metabolism and *Firmicutes* and *Gammaproteobacteria* populations were detected after ZYP treatment in both colitis and cholestasis. Therefore, we conclude that tryptophan metabolism and *Firmicutes* and *Gammaproteobacteria* populations are the core targets of the anti-inflammatory effect of ZYP. These findings provide a scientific basis for further investigation of the anti-inflammatory mechanism of ZYP in the future.

## Introduction

In recent years, with the change in diet patterns of the population, the incidence of diseases of the digestive system is gradually increasing (Van Cutsem et al., 2016). Colitis, which can lead to poor quality of life, weight loss, and malnutrition (Casellas et al., 2001; Saibeni et al., 2005; Hwang et al., 2012), has become a common clinical disease of the digestive system with an unknown etiology, affecting the health of individuals (Freeman et al., 2001; Colombel et al., 2017). According to statistical data, the incidence of colitis in southern China, such as Guangdong Province, has exceeded rates of 3/1,00,000 persons (Zeng et al., 2013). A long-term or chronic inflammatory response has been proven to be one of the important pathophysiological mechanisms in the progression of colitis (Okayasu, 2012; Onal et al., 2012), and corticosteroids are considered to be the most effective treatment for colitis. However, side effects and lack of benefit limit the clinical application of these treatment options (Varshosaz et al., 2011). Currently, the clinical treatment of colitis is in urgent need of potential effective drugs and new treatment regimens. In the thousands-of-years-old history of Traditional Chinese Medicine (TCM), many diseases have been treated. Therefore, TCM may inspire the clinical diagnosis and treatment of colitis.

Zhuyu pill (ZYP), a combination of *Coptis chinensis* Franch. (huanglian in chinese) and *Tetradium ruticarpum* (A. Jussieu) T. G. Hartley (Wuzhuyu in Chinese) in a ratio of 1:1, is a classic TCM prescription for treating diseases of the digestive system, such as cholestasis and colitis. It was traditionally used for the treatment of diarrhea and hematochezia in both Tai Ping Sheng Hui Fang (Wang, 2016) and Prescription Dictionary of Chinese Medicine (Peng, 1993), by its mechanism of regulating the balance of qi. In modern studies, the main active ingredients of ZYP, such as berberine and evodiamine, have been confirmed to have certain anti-inflammatory effects (Mohammadzadeh et al., 2017; Ding et al., 2020; Jiang et al., 2021) and can affect intestinal microecology by regulating bile acid metabolism (Wolf et al., 2021). Furthermore, interest in the lipid-lowering effect of the mixture of berberine and evodiamine in the 1:1 ratio has increased (Zhou et al., 2017), and the anti-Alzheimer’s effect of the two active ingredients has been reported (Fang et al., 2020). In our previous study, ZYP was confirmed to produce a significant anti-cholestasis effect by regulating fecal metabolism, fecal microbial diversity (Yu et al., 2021b), and miRNA expression (Yu et al., 2021a). The interventional effect of the Zuojin pill (ZJP, composed of *Coptis chinensis* Franch. and *Tetradium ruticarpum* (A. Jussieu) T. G. Hartley, usually mixed in a ratio of 6:1) on colitis has been the focus of attention in research (Zhou et al., 2020; Wei et al., 2021). These studies suggest that ZYP is a potential drug for the treatment of colitis. However, the interventional effect of ZYP on colitis has yet to be confirmed; the underlying biological mechanism remains unclear.

“Treating different diseases with the same treatment” is a unique original ideology in TCM (Zheng et al., 2015), which is based on the idea of treating different diseases with similar pathogenesis and clinical syndromes. Because both cholestasis and colitis have obvious inflammatory responses (Rosenberg et al., 2013; Zhang et al., 2019) and ZYP has potential therapeutic effects on both diseases, we propose that investigation of the common targets of the interventional effect of ZYP on cholestasis and colitis can elucidate the underlying mechanism of action of ZYP. To explore the common mechanism of action of ZYP in cholestasis and colitis from the perspective of fecal metabolism and fecal microbial diversity, we combined data from an experiment on the treatment of 2,4,6-trinitro-benzenesulfonic acid (TNBS)-induced colitis rat models with ZYP and data from previous studies (Yu et al., 2021b). The study sought to answer three questions. First, it sought to investigate whether ZYP has a clear mechanism of intervention in colitis rat model. Second, it sought to assess whether the dual effect of ZYP on fecal metabolism and fecal microbial homeostasis is the mechanism of action of ZYP in colitis rat model. Third, it aimed to assess the changes in biological function and bacterial number associated with the common mechanisms by which ZYP produces anti-inflammatory effects. This study provided a basis for further studies on the anti-inflammatory effect of ZYP, improved our understanding of “treating different diseases with the same treatment” ideology in TCM, and provided a potential drug for the clinical treatment of colitis.

## Materials and methods

### Reagents

TNBS was prepared using 5% TNBS (Sigma-Aldrich, St. Louis, MO, United States) and diluted to a final concentration of 2% TNBS and 50% ethanol with phosphate-buffered saline (PBS). *Coptis chinensis* Franch. and *Tetradium ruticarpum* (A. Jussieu) T. G. Hartley were purchased from Beijing Tongrentang Science and Technology Development Co., Ltd. (Beijing, China). L,-2-chlorophenylalanine was obtained from Shanghai Hengchuang Biotechnology Co., Ltd. (Shanghai, China). Water, methanol, acetonitrile, and formic acid were purchased from CNW Technologies GmbH (Düsseldorf, Germany). All chemicals and solvents were reagent grade or high-performance liquid chromatography (HPLC) analytical grade.

### Zhuyu pill preparation and quality control

ZYP was composed of two herbs. All compositions of ZYP are listed in Table 1.

**TABLE 1:** Composition of Zhuyu pill (ZYP).

Chinese name	Botanical name^a^	Plant family	Weight (g)	Part used	Herbal-producing region
Huanglian	*Coptis chinensis* Franch.	*Ranunculaceae*	6	Dried rhizome	Sichuan
Wuzhuyu	*Tetradium ruticarpum* (A. Jussieu) T. G. Hartley	*Rutaceae*	6	Dried ripe seed	Yunnan

^a^ The plant name has been checked with http://www.theplantlist.org.

This table was reproduced from (Yu et al., 2021a).

In previous studies, we used HPLC to detect the content of the main components of ZYP. As a result, ZYP contained 36.8 mg/g of berberine, 14.9 mg/g of coptisine, 0.78 mg/g of evodiamine, and 0.33 mg/g of rutecarpine. The fingerprints of different batches of ZYP decoction showed a consistent trend, indicating that ZYP has botanical characteristics (Yu et al., 2021a) (Supplementary Material S1). In this study, the preparation of ZYP was consistent with that of our previous studies (Yu et al., 2021a). Therefore, 120 ml of solution was extracted from 12 g of herbs; the final concentration of ZYP was 0.1 g/ml (w/v).

### Animals and treatments

Twenty-four male Sprague-Dawley rats aged 7–8 weeks, weighing 180 ± 20 g, were purchased from Chengdu Dossy Experimental Animals Co., Ltd. (Sichuan, China; certification no. SCXK-CHUAN 2020-030). All rats were housed under conventional conditions and had access to water and food *ad libitum*. Rats were acclimatized for 7 days prior to the experiments.

The rats were randomly allocated into the control, model, low-dose ZYP (ZYP_L), and high-dose ZYP (ZYP_H) groups. Each group consisted of six rats. Except the normal group, all groups were administered with a single TNBS enema to induce acute colitis (100 mg/kg) (Ji et al., 2020), 8 cm from the anus, using a catheter. The normal group was administered the same amount of vehicle (paroline) enema as the control. After the injection, the rats’ anuses were clamped, and the rats were maintained in a head-down position for 15 min. After TNBS induction, rats were administered ZYP (ZYP_L: 0.6 g/kg, ZYP_H: 1.2 g/kg) for 10 consecutive days, once a day *via* oral gavage. The control and model groups were given an equal volume of distilled water.

### Ethical approval

Ethics approval was granted by the Ethics Committee of Chengdu University of Traditional Chinese Medicine (Ethics Approval Number: 2019-15).

### Sample collection and intestinal injury evaluation

After the final ZYP administration, two fresh fecal pellets were collected from each rat. Fecal samples were stored frozen at −80°C until analysis.

After 17 days, rats were euthanized 16 h after the final ZYP administration. The distal 8-cm portion of the colon was excised immediately afterward. The colon length was measured with a ruler, and the colon was weighed. The weight-to-length ratio, representing the colon weight index (g/cm), was used to ascertain the degree of colon edema or retraction caused by inflammation.

As previously described (Cotter et al., 2018), disease activity index (DAI) is an effective indicator for evaluating colitis. In this study, body weight, stool viscosity, and bleeding status were recorded daily and scored with reference to the scoring standard (MacPherson and Pfeiffer, 1978) (Table 2). The DAI results were calculated according to the following equation, as reported in a previous study (Wang et al., 2021).

$$DAI=\frac{Percentage\;decrease\;in\;body\;mass+Stool\;consistency+Fecal\;bleeding\;status}{3}$$

**TABLE 2:** Scoring of disease activity index.

Weight loss	Stool consistency	Fecal occult blood	Score
Normal	Normal	Normal	0
1%–5%	Soft but still formed	Occult bleeding (+)	1
5%–10%	Loose stools	Occult bleeding (++)	2
10%–15%	Diarrhea	Occult bleeding (+++)	3
>15%	Diarrhea	Visible gross bleeding	4

### Histological analysis of colon injury and inflammatory response

At 4 cm from the anus, one colon segment (0.5 cm) was collected for histopathological analysis from all rats. All colonic samples were fixed in 4% paraformaldehyde, embedded in paraffin, and cut into 4–5 µm sections. After deparaffinization with xylene, the slides were further used for histopathologic (H&E) and immunohistochemical (tumor necrosis factor alpha, TNF-α; interleukin-1 beta, IL-1β; interleukin-4, IL-4) analyses. Slides were observed under an Eclipse E100 microscope (Nikon, Tokyo, Japan), and two images were obtained at ×100 and ×200 magnification for each field.

Colon damage index was evaluated based on colon histopathology scoring criteria (Table 3). Immunohistochemical analysis was performed using ImageJ (version 1.53a, National Institutes of Health, Bethesda, MD, United States).

**TABLE 3:** Colon damage index scoring criteria.

Score	Microscopic pathological change
0	Normal
1	Slight inflammatory cell infiltration
2	Mucosal epithelium was exfoliated with slight inflammatory infiltration
3	Mucosal epithelium was exfoliated and necrotic with severe inflammatory infiltration
4	Severe inflammatory infiltration; inflammatory cell infiltrates in the tunica mucosa and tunica submucosa
5	Extensive ulceration and transmural inflammation invaded the serosal layer

### Fecal metabolomics

A detailed protocol for this study has been previously described (Yu et al., 2021b). The extensive description of the metabolomics methods can be found in Supplementary Material S1. Raw sequence data were uploaded into the Metabolights database (https://www.ebi.ac.uk/metabolights/) and are available through accession number MTBLS4980.

### Fecal microbiota sequencing

The methods of microbiota sequencing were conducted, as described previously (Yu et al., 2021b). Extensive description of the metabolomics methods can be found in Supplementary Material S1. Raw sequence data were uploaded to the Sequence Read Archive (SRA) database (https://www.ncbi.nlm.nih.gov/) and are available through accession number PRJNA839869.

### Statistical analysis

Statistical analysis was performed using GraphPad Prism version 8 (GraphPad Software, Inc., San Diego, CA, United States). All experiments were replicated six times, and results are presented as the mean ± standard errors. One-way analysis of variance tests with *post-hoc* Tukey’s tests were used for comparisons among multiple groups. Statistical significance was set at *p* < 0.05.

## Results

### Therapeutic effects of Zhuyu pill on colitis

**FIGURE 1:**
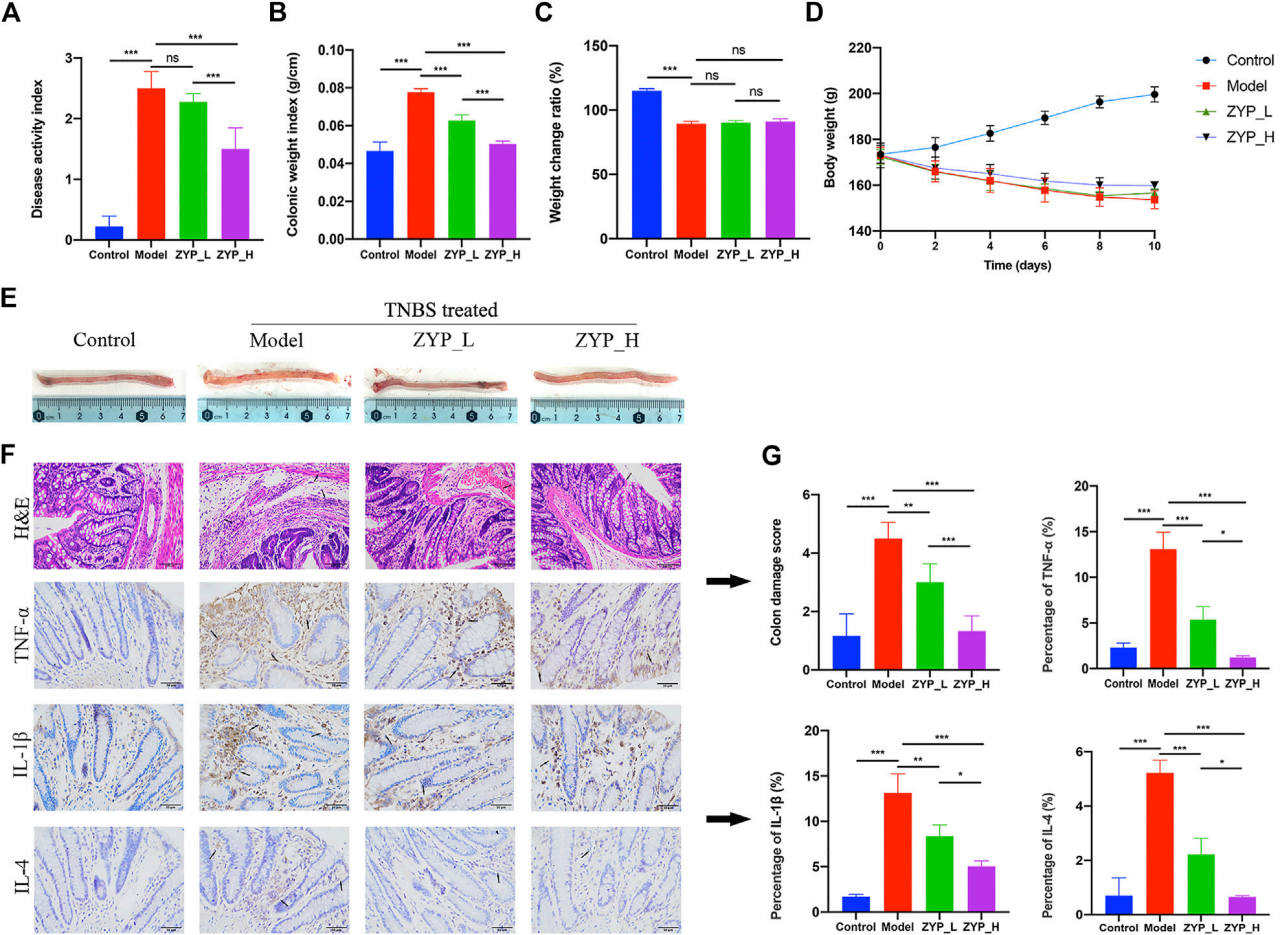
Evaluation of ZYP intervention effects. **(A)** DAI score; **(B)** Colonic weight index; **(C)** Weight change ratio; **(D)** Body weight change curves; **(E)** Colon phenotypes in the different groups; **(F)** Representative images of the H&E analysis and the TNF-α, IL-1β, and IL-4 immunohistochemical analysis; **(G)** Colon damage score and proportion of the TNF-α, IL-1β, and IL-4 cells in the four groups. Data are means ± SD, *n* = 6. ***/**/*, *p* < 0.001/*p* < 0.01/*p* < 0.05; ns, not significant.

Figure 1 shows the therapeutic effect of ZYP on colitis rats. According to Figure 1, TNBS significantly increased DAI score (Figure 1A) and colonic weight index (Figure 1B), reduced the body weight and weight change ratio of rats (Figures 1C,D), damaged colonic tissue structure, and increased the percentage of inflammatory cells in the colon (Figures 1E–G). Both low dose and high dose ZYP effectively alleviated these abnormal changes (except body weight and weight change ratio), and high dose ZYP had clearer intervention effect than low dose ZYP. In brief, ZYP has an intervention effect on colitis rats, and this effect has a dose-effect relationship.

### Multivariate statistical analysis of LC-MS

**FIGURE 2:**
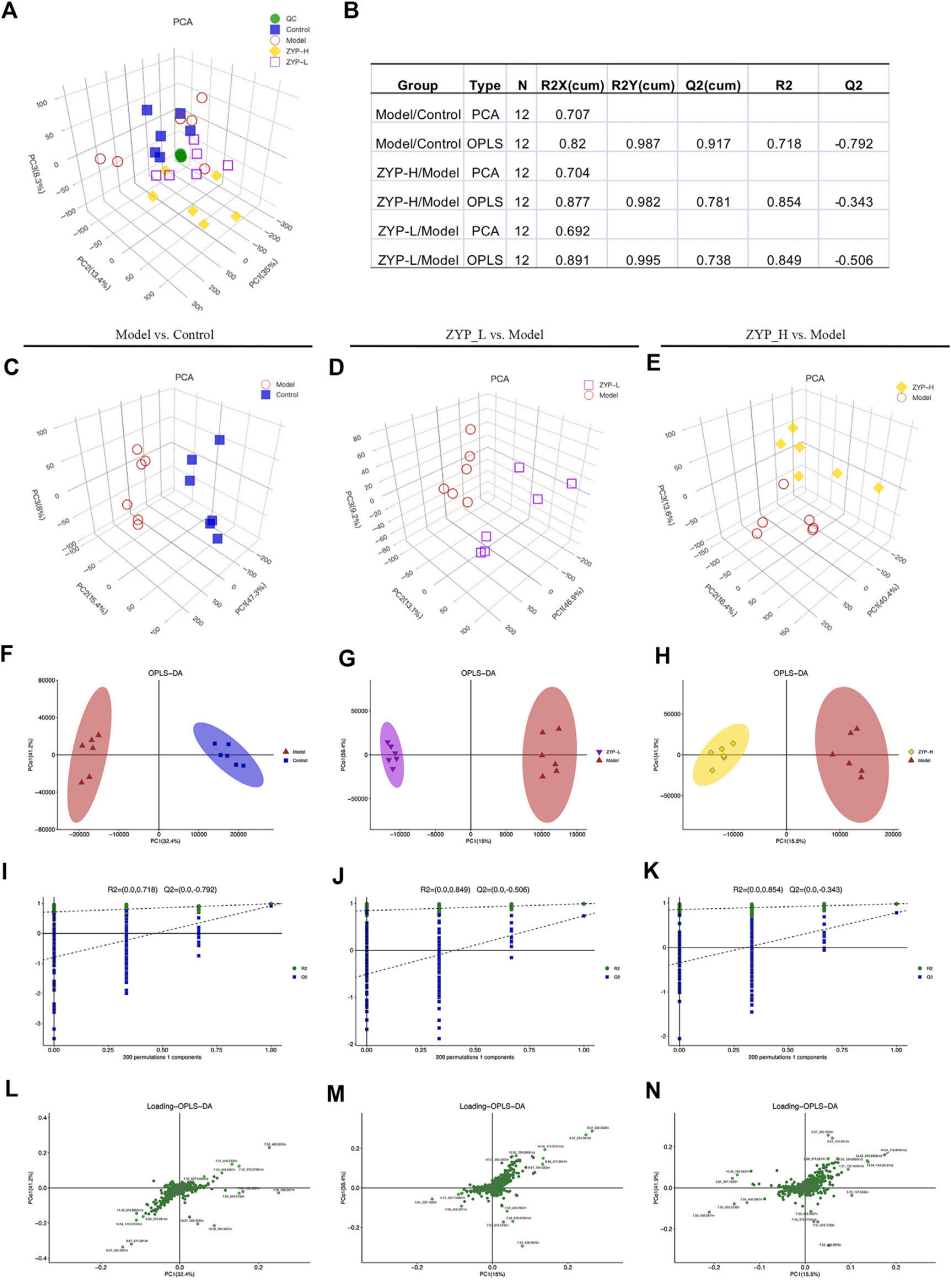
Multivariate statistical analysis of metabolic profiles derived from the control, model, ZYP_L, and ZYP_H groups. **(A)** PCA score plot of the control, model, ZYP_L, ZYP_H, and QC groups; **(B)** Parameters of PCA and OPLS-DA; **(C–E)** PCA analysis; **(F–H)** OPLS-DA analysis; **(I–K)** 200-permutation test; **(L–N)** OPLS-DA score plot.

PCA was used to compare the differences among all groups. As shown in Figure 2, a score plot allowed visualization of the observational clusters. Metabolic differences were observed among the control, model, ZYP_L, ZYP_H, and QC groups (Figures 2A,C–E). To further identify the metabolic differences among the groups, OPLS-DA analysis was performed. The difference between the Model and Control groups (R^2^X = 0.82, R^2^Y = 0.987, Q^2^Y = 0.917), ZYP_L and Model groups (R^2^X = 0.891, R^2^Y = 0.995, Q^2^Y = 0.738), and ZYP_H and Model groups (R^2^X = 0.877, R^2^Y = 0.982, Q^2^Y = 0.781) are shown in Figures 2B,F–H, these results indicate significant metabolic differences between control, model, ZYP_L, and ZYP_H groups.

To prevent overfitting, seven-cycle interactive verification and 200-response ranking test was applied. Based on these results, the OPLS-DA was not overfit and had a high separating capacity (Figures 2I–K). Furthermore, the loading scatter plot for OPLS-DA identified that few points had significantly drifted away from the measured center, indicating that these variables are important for clustering (Figures 2L–N).

### Metabolite identification and pathway analysis

VIP > 1 and *p* < 0.05 were set as criteria for differential metabolite screening. Consequently, 663 metabolites were significantly differentially expressed between the model and control groups (258 upregulated and 405 downregulated), whereas 408 and 492 metabolites were significantly differentially expressed between the ZYP_L and Model groups (281 upregulated and 127 downregulated) and ZYP_H and Model groups (275 upregulated and 217 downregulated), respectively (Supplementary Material S2).

**FIGURE 3:**
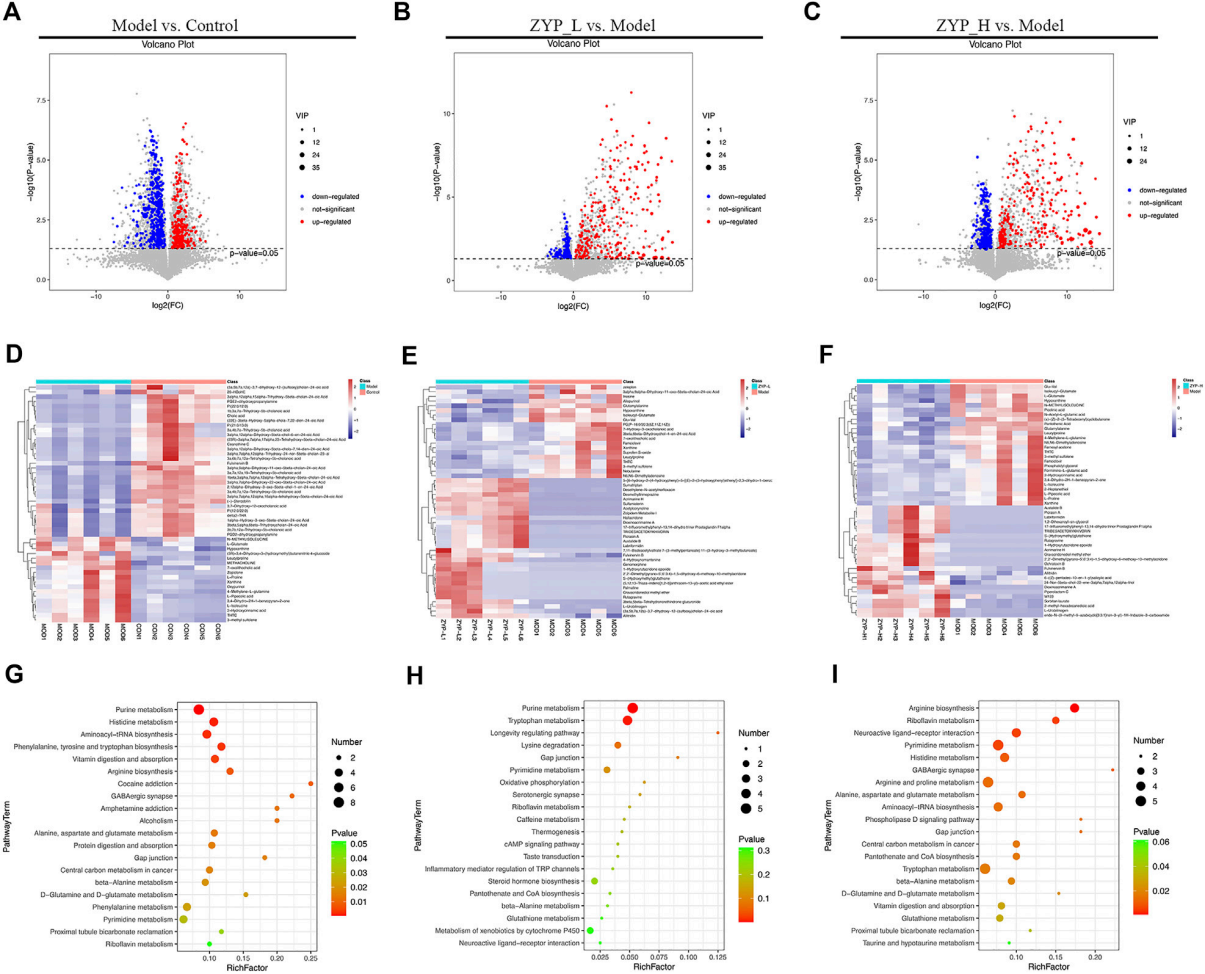
Volcano plot, heat map, and bubble diagrams of the control, model, and ZYP treatment groups. **(A–C)** Volcano plot of rat fecal metabolites. Each point represents a metabolite, and the point size represents the VIP value; **(D–F)** Heat map of rat fecal metabolites. Red represents a higher relative abundance, while blue illustrates a lower relative abundance; **(G–I)** Bubble diagrams of rat fecal metabolites. The color of each circle indicates the *p*-value, and the size of each circle reflects the varying metabolite number of each pathway.

Based on the results illustrated in Figure 1, a high dose of ZYP had a more significant interventional effect compared with a low dose of ZYP. Additionally, the dose administered to the ZYP_H group was the standard clinical dose of ZYP. Therefore, combined with the above reasons, understanding the metabolic differences between the ZYP_H and Model groups is scientifically significant. In the comparison between the ZYP_H and Model groups, 94 different metabolites belonged to the categories of amino acids, peptides, and analogues, suggesting that regulation of amino acid metabolism was one of the important mechanisms of the effect of ZYP on colitis. The metabolites with significantly differential abundance were observed through volcano plots (Figures 3A–C), and the heatmap showed the top 50 differential metabolites among the Control, Model, ZYP_L, and ZYP_H groups (Figures 3D–F).

To further analyze the mechanism of action of ZYP in colitis, the differential metabolites of Model vs. Control, ZYP_L vs. Model, and ZYP_H vs. Model were enriched by Kyoto Encyclopedia of Genes and Genomes (KEGG) pathway analysis, and the results were mapped unto a bubble graph (Figures 3G–I). As a result, low and high doses of ZYP significantly affected amino acid metabolism-related pathways such as glutathione metabolism and tryptophan metabolism in colitis.

Based on the “Treating different diseases with the same treatment” ideology, investigating the common effect of the TCM decoction on different diseases is an effective method for uncovering the actual effects of the compounds. Combined with our previous study on the effect of ZYP on fecal metabolism in cholestatic rats (accession number: MTBLS2721; https://www.ebi.ac.uk/metabolights/) (Yu et al., 2021b), four metabolic pathways were identified in the mechanism of ZYP on both cholestatic rats and UC rats. They included neuroactive ligand-receptor interaction, histidine metabolism, phospholipase D signaling pathway, and tryptophan metabolism. These metabolic pathways are thought to be key to the effect of ZYP in different inflammatory diseases and provide a valuable basis for further studies on the mechanism of ZYP in the future.

### Fecal microbiota analysis

To investigate whether the effect of ZYP is related to the changes in fecal microbiota, we analyzed the composition of fecal microbiota of rats after TNBS or ZYP treatment.

**FIGURE 4:**
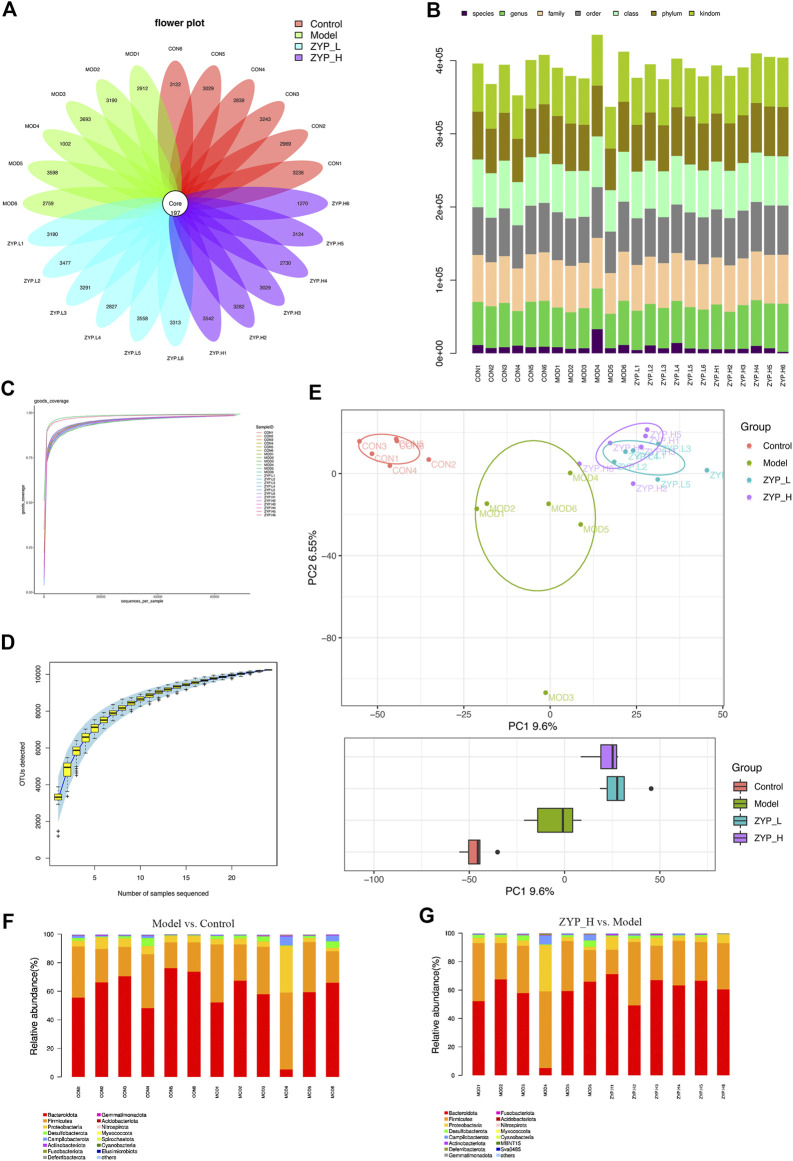
Classification of OTUs, alpha-diversity analysis, and beta-diversity analysis. **(A)** Operational taxonomic units (OTUs)-based petal maps. Each petal corresponds to a sample group, with the shared overlapping region representing OTUs common to all samples, and the numbers on individual petals representing the number of OTUs unique to a given sample group; **(B)** Comment situation of tags; **(C)** Curve of diversity index dilution; **(D)** Curve of SAC; **(E)** PCA analysis; **(F,G)** Relative abundance at phylum level.

In the OTUs-Venn diagram, 97% similarity of the OTUs was calculated using cluster tags (Figure 4A). Consequently, 197 OTUs were found in the Control, Model, ZYP_L, and ZYP_H groups. Information on species, genus, family, order, class, phylum, and kingdom for each sample are shown in Figure 4B.

To evaluate the rationality of sequencing quantity, alpha-diversity analysis was performed on different groups of samples in this study. The OTUs/diversity index dilution curve and Specaccum species accumulation curve showed that all curves tended to be flat eventually, indicating that the sampling volume and sequencing volume of this study were reasonable (Figures 4C,D).

Beta-diversity analysis was used to compare the diversity differences among different grouped samples. The representative results are presented in a PCA score plot, as shown in Figure 4E. PCA coordinates of the Control, Model, ZYP_L, and ZYP_H groups showed significant separations. Both TNBS and ZYP had significant effects on fecal microbial homeostasis.

In order to understand the composition of microbial communities, the relative abundance of all microbial communities was analyzed. It was observed that *Bacteroidota* (51.3%), *Firmicutes* (35.1%), and *Proteobacteria* (8.3%) were the most abundant species in the colitis model (Figure 4F). Similarly, after high dose ZYP intervention, the most abundant phyla were *Bacteroidota* (62.9%), *Firmicutes* (29.6%), and *Proteobacteria* (5%) (Figure 4G). These results indicated that ZYP significantly reduced *Firmicutes* abundance, elevated *Bacteroidetes* abundance, and lowered the ratio of *Firmicutes to Bacteroidetes.*

### Multivariate statistical analysis of microbial data

**FIGURE 5:**
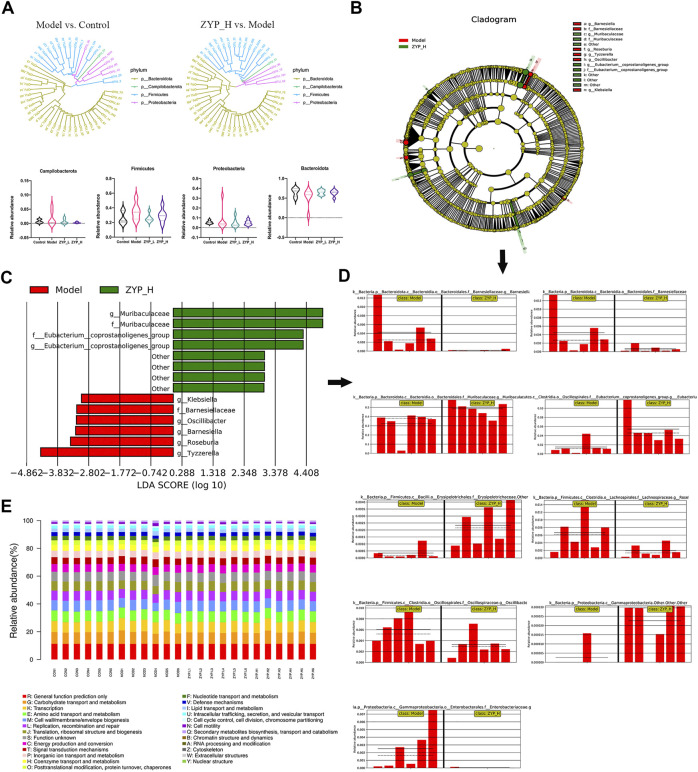
Multivariate statistical analysis of microbial data. **(A)** Phylogenetic analysis and histogram of relative abundance of top 50 OTUs. The phylogenetic tree was reconstructed using the maximum-likelihood method; **(B)** Evolutionary clade of LEfSe analysis, taxonomic representation of statistically and biologically consistent differences between model and ZYP_H groups. Differences are represented in the color of the most abundant class (red indicating model groups, green indicating ZYP_H groups); **(C)** Histogram of LDA value distribution (red indicating model groups, green indicating ZYP_H groups); **(D)** Relative abundance plots of representative microbiome; **(E)** Bar plot of COG analysis.

To further understand the effect of ZYP on the fecal microbes of colitis rats, a maximum of 50 OTUs with tags were selected for the phylogenetic analysis (Figure 5A)*.* To further understand which bacteria were different in the other groups, we conducted an LEfSe (linear discriminant analysis coupled with effect size measurements) analysis (Figures 5B–D). From the phylogenetic analysis in this study and our previous study (Supplementary Material S3, accession number: PRJNA720504; https://www.ncbi.nlm.nih.gov/) (Yu et al., 2021b), the relative number of *Firmicutes* and *Proteobacteria* were significantly affected by ZYP both in cholestasis and colitis. Furthermore, LEfSe results showed that the relative number of *Gammaproteobacteria* in both cholestasis (LDA = 4.2, *p* < 0.01) and colitis (LDA = 3.02, *p* < 0.05) rats was significantly affected by ZYP. These results suggest that these bacterial communities are the potential link of the common effect of ZYP on these diseases.

The Cluster of Orthologous Groups database (COG, http://www.ncbi.nlm.nih.gov/COG/) was used to predict the influence of different biological functions. As shown in Figure 5E, several biological pathways, including energy production and conversion, amino acid transport and metabolism, and lipid transport and metabolism, were predicted, suggesting that ZYP may have therapeutic effects on UC rats by its effect on these biological functions.

**FIGURE 6:**
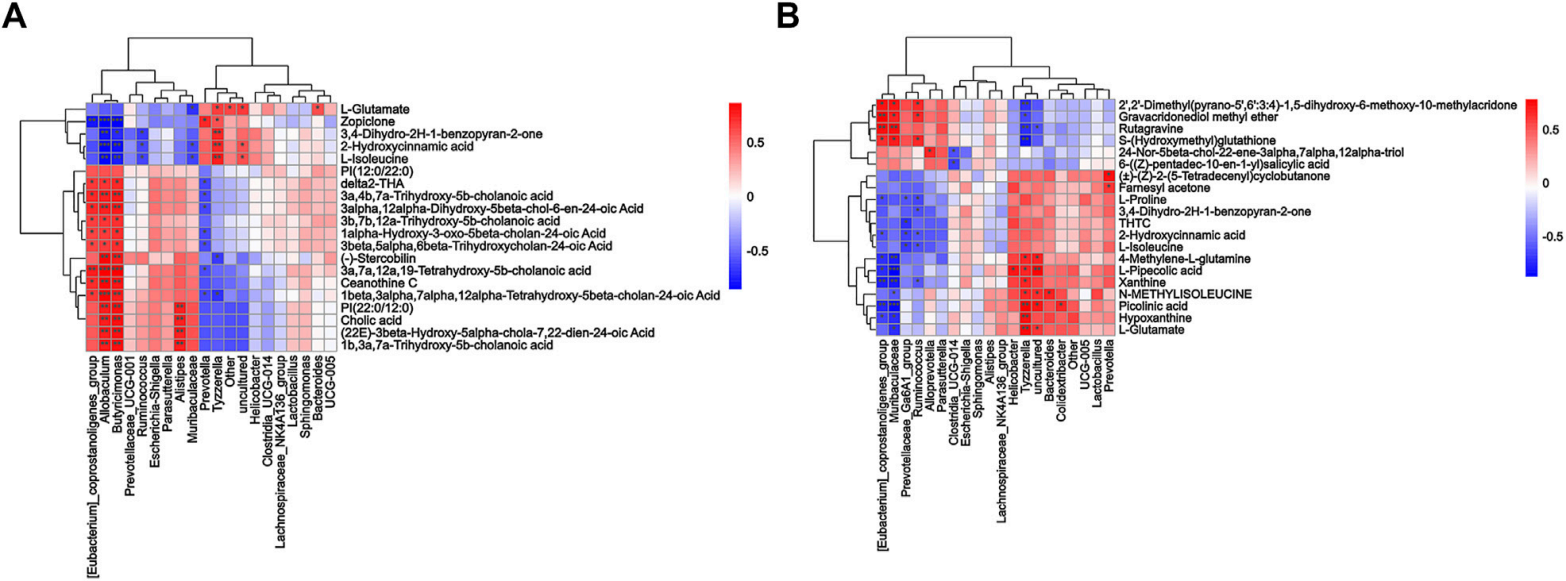
Correlation matrix network between fecal metabolites and genera of fecal microbiota. **(A)** Model vs. control; **(B)** ZYP_H vs. model. Correlation analysis of the results from the Oebiotech cloud platform (https://cloud.oebiotech.cn/task/detail/correlation-multiomics-oehw/), the color from red to blue represents the Spearman value from positive correlation to negative correlation. Asterisks represents significance of relevance: ***/**/*, *p* < 0.001/*p* < 0.01/*p* < 0.05.

To comprehensively analyze the relationships between fecal metabolites and genera of fecal microbiota, Spearman correlation analysis was performed for the Model vs. Control groups and ZYP_H vs. Model groups. A correlation matrix network was constructed. The paired correlations between the Model vs. Control groups and ZYP_H vs. Model groups indicated that there was a strong correlation between the fecal microbiota and fecal metabolites (Figure 6). The dual effect of ZYP on fecal microbiota and fecal metabolites is the potential mechanism of its therapeutic effect on colitis.

## Discussion

This study confirmed the interventional effect of ZYP on colitis using TNBS-induced colitis rat models. Using data from previous studies on the anti-cholestatic effect of ZYP, the study preliminarily discussed the common effect of ZYP on the two diseases from the perspective of fecal metabolism and fecal microbes.

DAI is the mean value of the stool consistency index, fecal bleeding index, and weight loss index. Colonic weight index is the colon weight-to-length ratio. According to previous studies, DAI, colonic weight index, and weight change ratio are well-known indicators of severity of colitis in rats (Shon et al., 2015; Zhang et al., 2016; Yu et al., 2017). The combination of these indicators can effectively evaluate the progression of colitis and degree of colonic edema from a macro perspective (Sun et al., 2017). The pathological observation of colonic tissue is an important index for evaluating the degree of colon injury, and the expression of inflammatory factors of the colon can effectively reflect the degree of inflammatory response of enteritis (Liu et al., 2020; Zhou et al., 2021). In this study, TNBS significantly increased the DAI index and colonic weight index of model rats, decreased the weight change ratio, and significantly damaged the colon histopathology, with severe inflammatory reactions. Both low and high doses of ZYP (0.6 and 1.2 g/kg/d) could effectively alleviate the abnormal expression of these indicators induced by TNBS. Except weight change ratio, high-dose ZYP (1.2 g/kg/d) had better interventional effect compared with low-dose ZYP (0.6 g/kg/d). Briefly, ZYP has a potential interventional effect on TNBS-induced colitis rats, and this interventional effect has a certain dose-response relationship.

Metabolomics is an important branch of systems biology. The comparative research methods of molecular biology, metabonomics, has the characteristic of integrity, which matches the whole concept and “multiple components, multiple targets, and multiple pathways” characteristics of TCM. In recent years, metabolomics has been an important research method for investigating the underlying mechanisms of prescription effects. In this study, 492 differential metabolites were screened in the comparison between the ZYP_H and Model groups, and the enrichment analysis of the above-mentioned differential metabolites was performed. According to previous studies, four metabolic pathways, including neuroactive ligand-receptor interaction, histidine metabolism, phospholipase D signaling pathway, and tryptophan metabolism, were affected by ZYP in both colitis and cholestasis models, suggesting that these pathways are potential targets of ZYP. According to previous studies, both colitis (Islam et al., 2017; Lanis et al., 2017; Yu et al., 2017) and cholestasis are associated with tryptophan metabolism disorders (Yu et al., 2018; Li et al., 2019). We found that these metabolic pathways are related to the tryptophan metabolism. First, the tryptophan metabolic pathway has been proven to play an important role in the treatment of diseases of the digestive system diseases. Tryptophan can reduce inflammation by regulating phospholipase (Liu et al., 2021). Second, neuroactive ligand receptor pathway involves several key substances, such as serotonin and glutamate receptors, that are present in the tryptophan metabolism pathways (Keever et al., 2020). The breakdown of tryptophan by kynurenines is accompanied by the production of several neuroactive intermediates (Ryabova et al., 2020). Third, serotonin and histamine are common mediators of inflammation, and tryptophan and histidine are important precursors for serotonin and histamine (Alfaifi et al., 2020). Particularly, in the theory of TCM, the abnormal rise and fall of *qi* can lead to a series of clinical manifestations, such as depression (Liao et al., 2017) and insomnia (Ye et al., 2015; Li et al., 2020), while the traditional use of ZYP on digestive system diseases is achieved by regulating *qi*. Coincidentally, the two main products in the tryptophan metabolic pathway, 5-HT and melatonin, are representative drugs for depression and insomnia (Cardinali et al., 2012). To some extent, the results of this study are consistent with the TCM theory. Based on these reasons, the regulation of tryptophan metabolism is probably one of the core mechanisms of ZYP in the treatment of colitis.

In recent years, several clinical (Maldonado-Arriaga et al., 2021) and experimental (Laubitz et al., 2008; Kiela et al., 2009; Lu et al., 2020) studies have reported the correlation between fecal microbes and colitis. Investigation of the number and diversity of fecal microbes is an effective way to study the pathogenesis of colitis. In our previous study, we confirmed the effects of high-dose ZYP (1.2 g/kg/d) on fecal microbial number and diversity in cholestatic rats, the phylogenetic analysis and histogram of relative abundance of the top 50 OTUs were shown as Supplementary Material S3. To find the common bacterial communities targeted by ZYP in colitis and cholestasis, the number and diversity of fecal microorganisms were assessed in this study. Three identical bacterial communities, *Firmicutes* (phylum level)*, Proteobacteria* (phylum level), and *Gammaproteobacteria* (class level), were obtained by combining the results of previous studies with those of this study. From the LEfSe analysis, *Roseburia, Tyzzerella, Eubacterium__coprostanoligenes, and Oscillibacter* belonged to the phylum *Firmicutes,* and *Gammaproteobacteria* belonged to the phylum *Proteobacteria.*

*Firmicutes* are an advantaged group of bacteria in the gastrointestinal tract and fecal flora of various animals (Ley et al., 2008). In inflammation, reduction of the ratio of *Firmicutes/Bacteroidetes* helps to inhibit the development of inflammatory reactions (Evans et al., 2014; Campbell et al., 2016). In addition, *Firmicutes* produce short-chain fatty acids that suppress inflammation to maintain intestinal health (Gophna et al., 2017). 5-HT/melatonin can significantly increase the levels of *Firmicutes* or decrease the ratio of *Firmicutes/Bacteroidetes* in both Parkinson’s disease and insomnia. Briefly, *Firmicutes* interact with tryptophan metabolism (Sun et al., 2018; Gao et al., 2020). *Gammaproteobacteria* have been considered to have differential effects at different stages of inflammatory response (Voorhies et al., 2019) and have certain correlations with the incidence of emotional diseases and intestinal 5-HT content (Zhuang et al., 2020). Briefly, these effects are essential for driving early inflammation necessary for protection against excessive inflammatory and autoimmune gastrointestinal disorders later in life (Mirpuri et al., 2014). Studies have found that the increase in the number of *Gammaproteobacteria* is related to reduced fatty liver (Michail et al., 2015; Guo et al., 2018), and the involvement of *Gammaproteobacteria* in amino acid transport and metabolism and lipid metabolism (Gifford et al., 2013; Scully et al., 2014) has been confirmed. In this study, high-dose ZYP significantly affected the number of *Firmicutes* and *Gammaproteobacteria* in both colitis and cholestasis, suggesting that these two bacterial communities are targets of ZYP in the treatment of inflammatory diseases. The biological functions of these bacterial communities were consistent with the functions associated with the findings from metabolomics enrichment. Additionally, the combined analysis of microorganisms and metabolites proved the existence of a relationship between relative number of microorganisms and relative metabolite level, indicating that changing the number of *Firmicutes* and *Gammaproteobacteria* can regulate amino acid metabolism and lipid metabolism pathways.

## Conclusion

ZYP is an effective drug combination used in TCM for the clinical treatment of diseases of the digestive system. Using LC-MS and 16S rRNA gene sequencing, we explored the changes in fecal metabolism and fecal microorganisms in rats with colitis and the effect of ZYP. Consequently, ZYP had a significant interventional effect on colitis and had a dual effect on fecal metabolic homeostasis and fecal microbial number. Furthermore, based on the “treating different diseases with the same treatment” ideology of TCM, we synthesized our results and those of previous studies and found that ZYP has an effect on tryptophan metabolism and *Firmicutes* and *Gammaproteobacteria* populations in both colitis and cholestasis models. The changes in biological functions and bacterial population may be the core mechanism of the interventional effect of ZYP in different inflammatory diseases. These findings provide an effective scientific basis for further investigation of the anti-inflammatory mechanism of ZYP in the future.

## Data Availability

The datasets presented in this study can be found in online repositories. The names of the repository/repositories and accession number(s) can be found below: https://www.ncbi.nlm.nih.gov/, PRJNA839869; https://www.ebi.ac.uk/metabolights/, MTBLS4980.

## Abbreviations

DAI, disease activity index; GO, gene ontology; HE, hematoxylin-eosin; HPLC, high-performance liquid chromatography; IL-1β, interleukin-1 beta; IL-4, interleukin-4; KEGG, kyoto encyclopedia of genes and genomes; OPLS-DA, orthogonal partial least-squares-discriminant analyses; PCA, principle component analysis; TCM, traditional Chinese medicine; TNBS, 2,4,6-trinitro-benzenesulfonic acid; TNF-α, tumor necrosis factor alpha; ZYP, Zhuyu pill; ZYP_H, high-dose ZYP; ZYP_L, low-dose ZYP.
