# Supplementary figures and images for: Mechanism of interventional effect and targets of Zhuyu pill in regulating and suppressing colitis and cholestasis
Source: Front Pharmacol. 2022 Nov 2;13:1038188. doi: 10.3389/fphar.2022.1038188 (PMC9666482; doi:10.3389/fphar.2022.1038188)

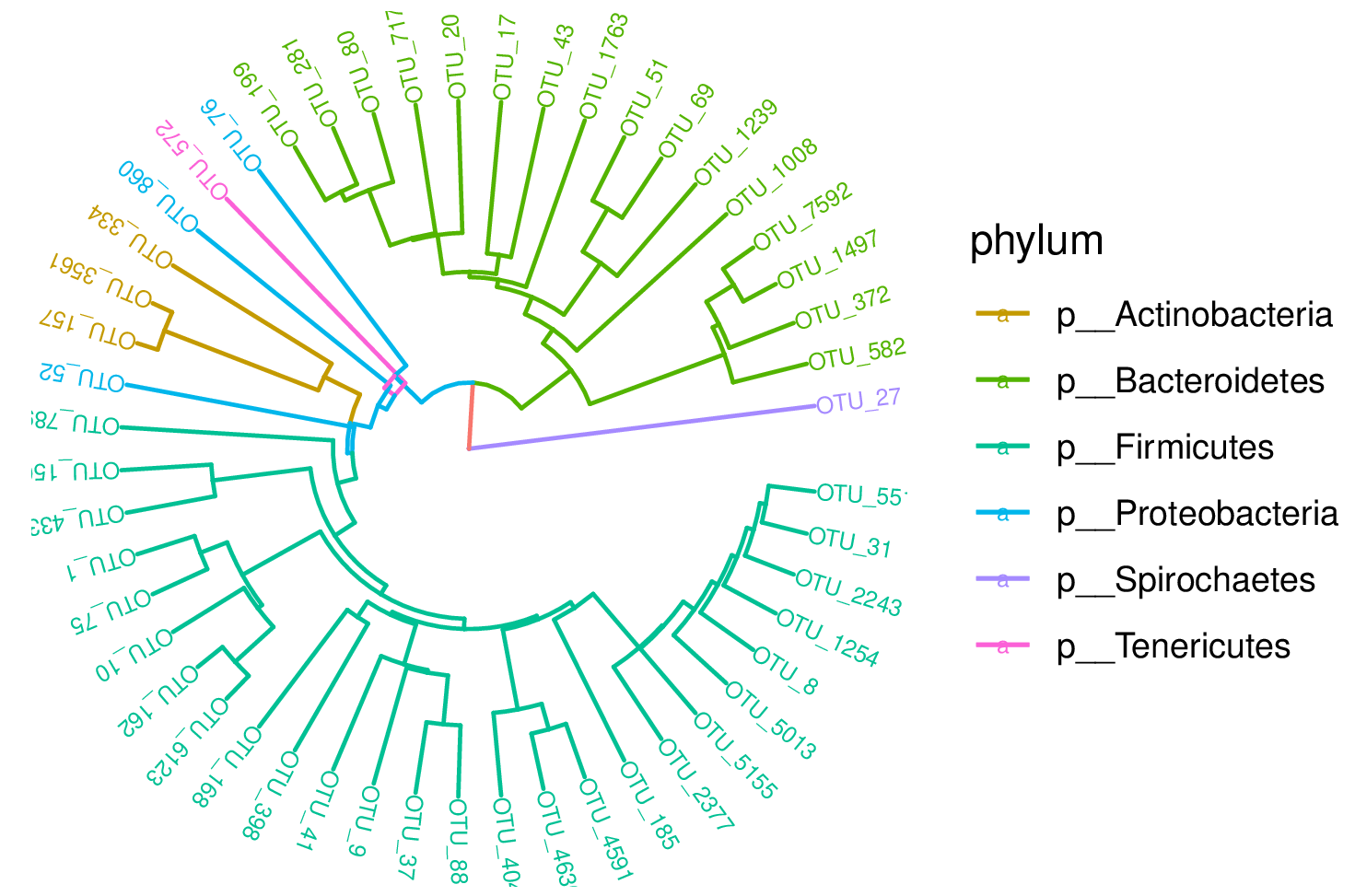

Supplement: Supplementary file 1 [file Image1.PNG]
