# Supplementary material for: Mechanism of interventional effect and targets of Zhuyu pill in regulating and suppressing colitis and cholestasis
Source: Front Pharmacol. 2022 Nov 2;13:1038188. doi: 10.3389/fphar.2022.1038188 (PMC9666482; doi:10.3389/fphar.2022.1038188)
Supplement: Supplementary file 2 [file DataSheet1.docx]

*ZYP Preparation and Quality Control*

In this study, the ZYP decoction was prepared by boiling the herbs in water twice. For every 240 ml of water, there were 6 g each *of Coptis chinensis Franch.* and *Tetradium ruticarpum (A. Jussieu) T. G. Hartley.* The mixture was heated at 100°C for 45 min, filtered, and the suspension was collected for later use. The filtered crude herbs were then added to another 240 ml of water, heated at 100°C for 45 min, filtered, and the suspension was collected. The two suspensions were mixed thoroughly and concentrated to 120 ml using a rotary evaporator. As a result, 120 ml of solution was extracted from 12 g of crude Chinese herbs, making the final concentration of ZYP 0.1 g/ml (w/v). The extraction solution was stored at -20°C before administration.


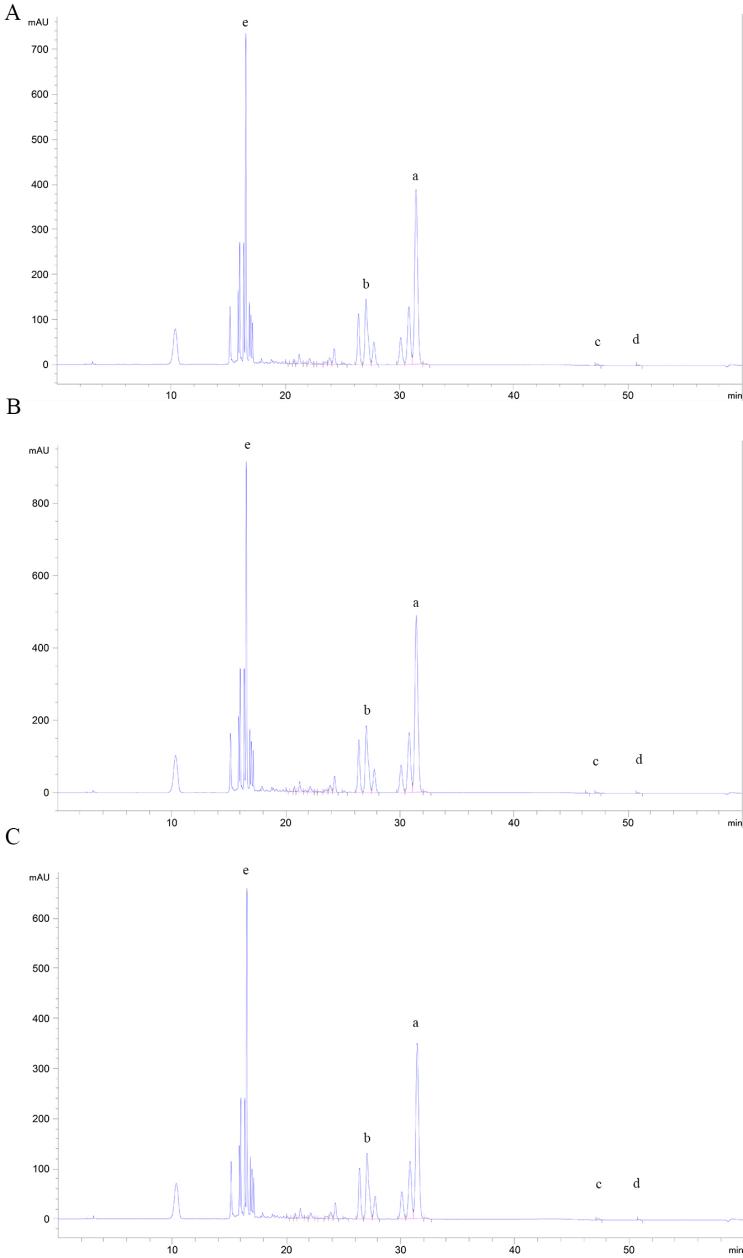


**Fingerprint of the Zhuyu pill and its formulae.** (A-C) Three ZYP technical repetitions in HPLC analyses. a) berberine; b) coptisine; c) evodiamine; d) rutaecarpin; e) other unknown materials.

*Sample Preparation for Metabolome Profiling and LC-MS analysis*

A 60 mg stool sample was accurately weighed and transferred to a 1.5-mL Eppendorf tube. Two small steel balls were added to the tube. A 20 μL volume of internal standard (2-chloro-l-phenylalanine in methanol, 0.3 mg/mL) and 600 μL extraction solvent with methanol/water (4/1, v/v) were added to each sample. Samples were stored at -20℃ for 5 min and then grinded at 60 Hz for 2 min, ultrasonicated at ambient temperature (25℃ to 28℃) for 10 min, stored at -20℃ for 30 min. The extract was centrifuged at 9800 × *g*, 4℃ for 10 min and then, 300 μL of the supernatant in a brown and glass vial was dried in a freeze concentration centrifugal dryer. Next, 400 μL of a methanol and water mixture (1/4, v/v) was added to each sample, samples vortexed for 30 s, ultrasonicated for 3 min, and then placed at -20℃ for 2 h. Finally, samples were centrifuged at 9800 × *g*, at 4℃ for 10 min, and the supernatants (150 μL) from each tube were collected using crystal syringes, filtered through 0.22-μm microfilters, and transferred to LC vials. The vials were stored at -80°C until LC-MS analysis. QC samples were prepared by mixing aliquots of all samples to form a pooled sample. All extraction reagents were precooled at -20℃ before use.

*LC-MS Analysis*

A Dionex Ultimate 3000 RS UHPLC system fitted with Q-Exactive quadrupole-Orbitrap mass spectrometer equipped with heated electrospray ionization (ESI) source (Thermo Fisher Scientific, Waltham, MA, USA) was used to analyze the metabolic profiles in both the ESI positive and ESI negative ion modes. An ACQUITY UPLC HSS T3 (100 mm×2.1 mm, 1.8 μm) was employed in both positive and negative modes. The binary gradient elution system consisted of (A) water (containing 0.1 % formic acid, v/v) and (B) acetonitrile (containing 0.1 % formic acid, v/v) and separation was achieved using the following gradient: 5–20 % B over 0–2 min, 20–60 % B over 2–4 min, 60–100 % B over 4–11 min, the composition was held at 100 % B for 2 min, then 13–13.5 min, 100 % to 5 % B, and 13.5–14.5 min holding at 5 % B. The flow rate was 0.35 mL/min and column temperature was 45 ℃. All the samples were kept at 4℃ during the analysis. The injection volume was 2 μL.

The mass range was from m/z 100 to 1000. The resolution was set at 70,000 for the full MS scans and 17,500 for MS/MS scans. The collision energy was set at 10, 20, and 40 eV. The mass spectrometer operated as follows: spray voltage, 3800 V (+) and 3000 V (−); sheath gas flow rate, 35 arbitrary units; Aux gas flow rate, 8 arbitrary units; capillary temperature, 320°C.

The QCs were injected at regular intervals (every 8 samples) throughout the analytical run to provide a set of data from which repeatability could be assessed.

*Data Preprocessing and Statistical Analysis*

The acquired LC-MS raw data were analyzed using the Progenesis QI software (version 2.3, Nonlinear Dynamics, Newcastle, UK), based on a self-built databases. It also includes information on metabolite structure and mass spectrum data to address metabonomics biology. The following parameters were used: precursor tolerance was set 5 ppm, product tolerance was set 10 ppm, and retention time (RT) tolerance was set 0.02 min. Internal standard detection parameters were deselected for peak RT alignment, isotopic peaks were excluded for analysis, and the noise elimination level was set at 10.00, the minimum intensity was set to 15% of the base peak intensity. An Excel file was obtained containing three-dimensional data sets including m/z, peak RT, and peak intensities, and RT–m/z pairs were used as the identifier for each ion. The resulting matrix was further reduced by removing any peaks with missing value (ion intensity = 0) in more than 50% of the samples. Positive and negative data were combined data, which was then imported into R ropls package.

Principle component analysis (PCA) and Orthogonal partial least-squares-discriminant analyses OPLS-DA were carried out to visualize the metabolic alterations between experimental groups, after mean centering (Ctr) and Pareto variance (Par) scaling, respectively. The Hotelling’s T2 region, shown as an ellipse in the score plots of the models, defined the 95% confidence interval (CI) of the modeled variation. Variable importance in the projection (VIP) ranked the overall contribution of each variable to the OPLS-DA model, and variables with VIP >1 were considered relevant for group discrimination. Default seven-round cross-validation and the 200-response permutation test were applied, with one-seventh of the samples excluded from the mathematical model in each round to avoid overfitting.

The differential metabolites were selected based on the combination of a statistically significant threshold of variable influence on projection (VIP) values obtained from the OPLS-DA model and *P*-values derived from a two-tailed Student’s t test using the normalized peak areas, where metabolites with VIP>1.0 and *P*<0.05 were considered as differential metabolites.

**DNA Extraction, Library Construction, and Sequencing**

Total genomic DNA was extracted using DNA Extraction Kit following the manufacturer’s instructions (Cat.12888, QIAGEN, Dusseldorf, Germany). The DNA concentration was verified with using the NanoDrop and by agarose gel electrophoresis. The DNA genome was used as template for PCR amplification with barcoded primers and Tks Gflex DNA Polymerase (R060B, TaKaRa Bio, Beijing, China). For bacterial diversity analysis, the V3–V4 variable region of the 16S rRNA gene was amplified using primers 343F and 798R, using a commercial PCR kit (Cat. 51531, Qiagen).

Amplicon quality was visualized by gel electrophoresis, and amplicons were purified with AMPure XP beads (Cat. A63880, Agencourt), and amplified for another round of PCR. After a second purification with the AMPure XP beads, the final amplicon was quantified using Qubit dsDNA assay kit (Cat. Q32854, Thermo Fisher Scientific). Equal amounts of purified amplicon were pooled for subsequent sequencing based on Novaseq PE250.

**Taxonomical Annotation**

Raw sequencing data were exported in FASTQ format. Paired-end reads were then preprocessed using Trimmomatic software to detect and eliminate ambiguous bases (N). Further, low quality sequences were eliminated having an average quality score below 20 using sliding window trimming approach. After trimming, paired-end reads were assembled using FLASH software (version 34.0.0.118). Parameters of assembly were: 10 bp of minimal overlapping, 200 bp of maximum overlapping and a 20% maximum mismatch rate. Sequences were further filtered as follows: reads with ambiguous, homologous sequences or below 200bp were abandoned; reads with 75% of bases above Q20 were retained; and, reads with chimera were detected and removed. These steps were achieved using QIIME software (version 1.8.0).

Clean reads were subjected to primer sequences removal and clustering to generate operational taxonomic units (OTUs) using Vsearch software (Version 2.4.2) with a 97% similarity cutoff. The representative read of each operational taxonomic unit (OTU) was selected using the QIIME package. All representative reads were annotated and blasted against Unite database (ITSs rDNA) using pynast (v0.1).
